# Phytochemical Constituents, Folk Medicinal Uses, and Biological Activities of Genus *Angelica*: A Review

**DOI:** 10.3390/molecules28010267

**Published:** 2022-12-28

**Authors:** Gaber El-Saber Batiha, Hazem M. Shaheen, Esraa A. Elhawary, Nada M. Mostafa, Omayma A. Eldahshan, Jean-Marc Sabatier

**Affiliations:** 1 Department of Pharmacology and Therapeutics, Faculty of Veterinary Medicine, Damanhour University, Damanhour 22511, Egypt; 2 Department of Pharmacognosy, Faculty of Pharmacy, Ain Shams University, Cairo 11566, Egypt; 3 Center for Drug Discovery Research and Development, Ain Shams University, Cairo 11566, Egypt; 4 Institut de Neurophysiopathologie (INP), CNRS UMR 7051, Faculté des Sciences Médicales et Paramédicales, Aix-Marseille Université, 27 Bd Jean Moulin, 13005 Marseille, France

**Keywords:** *Angelica*, Umbellifereae, coumarins, biological activity, phytochemistry, traditional uses

## Abstract

Genus *Angelica* is one of the widely distributed and well-known genera of family Umbelliferae. It is utilized mainly by Chinese and Korean populations especially in their folk medicine. *Angelica* comprises a lot of medicinally important phytoconstituents such as coumarins, furanocoumarins, flavonoids, essential oils, verbascosides, polysaccharides, *etc*. Members of this genus play important roles, namely antioxidant, anti-inflammatory, anti-microbial, anti-diabetic, skin-whitening, cytotoxic, hepatoprotective, and many others. This review draws attention to many species of genus *Angelica* with much focus on *A. dahurica* being one of the highly medicinally used species within this genus.

## 1. Introduction

**Figure 1:**
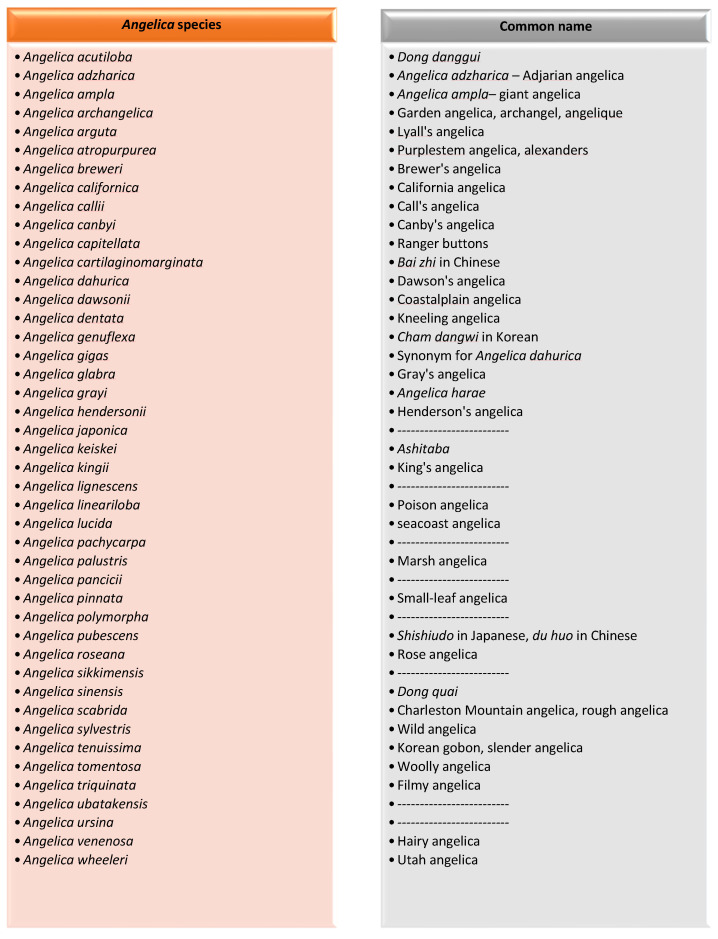
Diagram showing different *Angelica* species.

Family Apiaceae (Umbelliferae), also known as the carrot family, is one of the largest plant families. It is composed of 466 genera and about 3800 species that are distributed worldwide (Figure 1). Many members of this family are well-known by people from different cultures due to their odors, flavors, or toxicity. The most famous members are anise, fennel, cumin, caraway, dill, parsley, coriander, *etc*. The root parts of the family members play an important role in the medical field, and the most popular among them are: carrots (*Daucus carota*), celeriac (*Apium graveolens*), parsnips (*Pastinaca sativa*), angelica (especially *Angelica dahurica*), parsley root (*Petroselinum crispum*), alexanders (*Smyrnium olusatrum*), pignut (*Conopodium majus*), and skirret (*Sium sisarum*) [1].

Genus *Angelica* is a member of family Apiaceae (Umbelliferae), which is composed of 60 to 90 species of biennial perennial herbs that are widely distributed in Asia, Europe, and North America. Forty-five species out of 90 are located in China (about 32 endemic species) [2]. Plants of the genus *Angelica* are known as “women’s ginseng” in Southwest Asia and are used to treat amenorrhea and dysmenorrhea, menopausal disorders, hypertonia, anemia, and vascular dystonia; in many countries, these plants are accepted as officinal [3,4]. *Angelica dahurica* is considered one of the most important species of genus *Angelica,* especially its roots. Various phytoconstituents had been isolated from *A. dahurica* roots *viz.* coumarins, furanocoumarins, phthalides, polysaccharides, benzofurans, alkaloids, phenols, and sterols.

The dried root of *A. dahurica* is known as ‘Bai Zhi’ (*Angelica dahurica*; named Chuan Baizhi, Yu Baizhi, and Qi Baizhi), an important herbal medicine documented in the Chinese pharmacopoeia. It was reported that the pharmacological activities of this natural herb include protection against dexamethasone-induced ailments, hepatoprotection, antimicrobial effect, and anti-inflammatory and cytotoxic activities [5]. Several coumarins isolated from *A. dahurica* have been extensively studied for their chemical structure [6,7] and pharmacological effects [8,9].

*Angelica dahurica* root has been widely used for the treatment of acne, erythema, sinusitis, cold, headache (especially for migraine), toothache, and even cancer, for decades in Asia [10,11]. The *A. dahurica* root is also used for leukorrhea and arthralgia due to wind-dampness in Korean traditional medicine [12]. The root of the same species helps also in the protection against sepsis [13], and has anti-staphylococcal activity [10,14].

Northern *Angelica* (*Angelica archangelica* or *Angelica officinalis*) is widely used nowadays, both in the folk medicine and officinal medicine of many countries. In addition to *A. archangelica*, *A. dahurica*, *Angelica ursina*, *Angelica sylvestris,* and a range of other species are used for the preparation of medicines [15].

Huato Boluses containing *Angelica sinensis* and *Angelica dahurica* roots have gained great popularity during the recent years. The high antioxidant activity of ethereal oil from the roots of plants belonging to the genus *Angelica* has been reported [16,17,18].

This review work aimed at providing comprehensive and updated literature data on genus *Angelica* and its common species *A. dahurica,* based on published research articles, emphasizing the phytochemistry, biological activities, folk medicinal uses, potential toxicity, and side effects. In addition to that, this review may help researchers around the world find more information on genus *Angelica,* which will provide new research opportunities regarding the understudied phytochemical constituents and biological activities of this genus. This review covers the reported literature dating back to 2000, until 2022. The data collected in this review were summarized from different research databases *viz.* Scopus, Google Scholar, and PubMed.

### 1.1. Distribution

Today, *A. sinensis* is cultivated widely in the Gansu, Yunnan, Sichuan, Shaanxi, and Hubei provinces of China. It is also cultivated in Vietnam as a medicinal plant [19].

In most cultivation areas, *A. sinensis* seeds are placed in June and harvested in October of the next year. However, in Hubei Province and Zhanyi county, Yunnan Province, where the climate is much warmer and the altitude of the cultivated regions is much lower, growth is faster than in other regions, so it is sown in January and harvested in December of the same year. Post-harvesting, the soil is then rinsed off, and the rootlets and stalk are trimmed, after that, the roots are air-dried, then grouped into 0.5–1 kg flat bundles and baked dry over a slow fire [20]. *A. gigas* Nakai is located in Korea and usually used as *Angelica* roots in Southeast Asia [21].

### 1.2. Morphology

Genus *Angelica* carries the same general morphological characteristics of family Umbelliferae. The members of family Umbelliferae are usually annual or perennial herbs and rarely woody at their bases. The stems are caulescent or acaulescent, and either solid or hollow. The leaves are alternate, compound, pinnate, petiolated with sheath at the base of the petiole, and exstipulate. Leaflets are lanceolate in shape. Flowers are epigynous, small with long pedicels. The fruits are dry with two mericarps that unite to form one cremocarp, and each mericarp is one-seeded (Table 1) [1,22].

*Angelica dahurica* produces white flowers that bloom in umbrella-like clusters in June and July. The plant typically grows to a height of approximately 2 m (Table 1). The dried root is valued for its therapeutic properties. Its flavor is a distinct blend of bitter, sweet, and pungent, and its overall effect is warming in nature [23].

*Angelica sinensis* (Oliv.) Diels is a perennial plant, 0.4–1 m tall. The root is cylindrical, branched, succulent, strong aromatic, with many rootlets. The stem is ribbed, and is branched from above. The leaflets are ovate or ovate-lanceolate, with serrate margins. The flowers have 13–36 umbellules. The petals are white in color, and sometimes purplish-red in color. The fruits are ellipsoid or suborbicular (4–6.9, 3–4 mm) (Table 1) [24,25].

*Angelica archangelica,* also known as garden angelica, is a biennial herbaceous species of genus *Angelica.* It has a hollow stem carrying compound, alternate, yellowish-green leaves. It blooms during the summer, the seeds ripen in late summer, and then the plant dies. The flowers are greenish-white in color and small in size [26].

*A. gigas* is a stout plant that is 1 to 2 m high, with deep thick roots, and a purplish, ribbed stem. It has deeply dissected, very big, broad, pointy leaves. The plant is a biennial that flowers in the months of July to August in dark purple umbels, and self-seeds abundantly when the seeds have ripened [27].

*A. glauca* is an erect perennial plant of the temperate and alpine regions of the Himalayas. It is herb, about 1–2.5 m tall, glabrous, and having a stout stem. The roots are tuberous, thick, and aromatic. The leaves are petiolate, compound, and showing 1–2-ternate-pinnate venation; the leaflets are oval to ovate, mucronate-serrate, and glaucous. The flowers are usually bisexual, pedicellate, epigynous, actinomorphic, with pentamerous whorls and compound umbel inflorescence. The flower petals are free, five in number, white colored, and obovate in shape. The stamens are also five in number, green-coloured, bilobed, dorsifixed, exerted, and alternate to petals. The fruits are oblong-ellipsoid with prominent dorsal ribs [28].

*A. acutiloba* grows to about 0.3–1 m high. The color of the stems ranges from reddish to purplish. The stems are erect, glabrous, and thinly ribbed. The leaves are deep green, and alternately arranged, often with a leathery or fleshy texture. In most cases, the lower and basal leaves are petiolate or perfoliate. The petioles attached to them are about 10–30 cm in length. The mature blades are one- or two-pinnatifid. Young blades are usually three-pinnatifid. The leaves are of variable sizes. The upper leaves are simplified to oblong, with lanceolate and dentate incised blades. The leaf lobes are about 2–9 cm long and 1–3 cm wide. Most leaves are sessile, but sometimes they bear short stalks [29].

### 1.3. Traditional Uses

*Angelica dahurica* Radix (ADR; ‘Baek-ji’ in Korean, ‘Bai-zhi’ in Chinese) is commonly used in the traditional Korean and Chinese pharmacopoeias, such as Gumiganghwal-tang and Oyaksungi-san. In traditional Oriental medicine, *Angelica dahurica* was reported to be used as an anti-inflammatory agent for respiratory diseases (e.g., common cold and nasal congestion), dermal disorders (e.g., acne, ulcer, and carbuncle), pain (e.g., headache, toothache, and rheumatism), and intestinal disorders (e.g., diarrhea, dysentery, and chronic ulcerative colitis) [30,31,32].

In traditional Chinese medicine (TCM), *A. sinensis* was reported for the treatment of various diseases such as gynecological diseases, apoplexia, constipation, malaria, chills, fever, and hemorrhoids. The plant has also been used as a supplement in anemia as a haematopiotic agent, to regulate menstrual cycles, and to relax the bowels in constipation [33,34].

In traditional Korean medicine, *A. gigas* roots have been used for anemia, gynecological disorders, cardiovascular diseases, arthritis, sedative, analgesic, and tonic agents [11,35,36,37]. *A. acutiloba* is traditionally used to treat gynecological diseases and anemia [38].

Nevertheless, *A. archangelica* was traditionally valued in curing nervousness, insomnia, stomach and intestinal disturbances, skin diseases, respiratory problems, and arthritis [39], while *A. glauca* was used to treat bilious complaints, infantile atrophy, and constipation [40]. Moreover, *A. dahurica* was used to treat headaches, rhinitis, toothaches, rheumatism, and sore throat [41], and *A. pubescentis* was used to treat rheumatoid arthritis, headache, paralysis, and insomnia [42,43].

“Dang-Gui” refers to a raw drug belonging to the genus *Angelica* (Umbelliferae) that has been widely used as a traditional medicinal plant throughout Korea, China, and Japan. Dang-Gui carried different botanical names in three pharmacopoeias (Korean, Chinese, and Japanese), namely *Angelica gigas* Nakai, *Angelica sinensis* (Oliv.) Diels, and *Angelica acutiloba* Kitagawa, respectively. In the Korean pharmacopoeia, the roots of *A. gigas* are (“Dang-Gui”), while those of *A. acutiloba* are (“Il-Dang-Gui”) or the Japanese Dang-Gui [44,45].

### 1.4. Toxicity and Side Effects of Genus Angelica

Although many members of family Umbelliferae are edible fruits, condiments, and flavouring agents, members of genus *Angelica* may present certain side effects and toxicity from their use. Furanocoumarins from *A. archangelica,* especially the linear furanocoumarin, 8-methoxypsorlan, may present serious skin reactions and phototoxicity, especially in the presence of UV light [46]. Moreover, abdominal pain, convulsions, elevated bilirubin level, diarrhea, dystonia, and GIT hemorrhage were associated with *A. sinensis* root use [47]. Concerning *A. dahurica*, fewer reports were found on its potential toxicity; however, one report mentioned an LD_50_ value of (55–89 g/Kg) in mice, suggesting less toxicity [48].

## 2. Effects of Drying Methods on Contents of Bioactive Compounds and Antioxidant Activities of *Angelica dahurica*

Liang et al. (2018) aimed to analyze the quality (antioxidant and furanocoumarin content) of Bai Zhi roots after freeze-drying (the control), and in-the-shade, 40, and 70 °C drying. Antioxidant activity was evaluated through DPPH and iron-chelating assays. Six furanocoumarin compounds *viz.* xanthotoxin, bergapten, oxypeucedanin, imperatorin, phellopterin, and isoimperatorin were detected by HPLC. Shade-dried roots showed higher antioxidant activity compared to (40 and 70 °C) drying and freeze-drying. The furanocoumarin content was similar for both 70 °C drying and freeze-drying. Thus, it was concluded that *A. dahurica* roots dried at 70 °C may be an alternative method for maintaining high quality [49,50].

## 3. Phytochemical Constituents

Different phytochemical constituents belonging to diverse chemical classes had been reported from genus *Angelica*. The reported phytochemicals included mainly essential oils, coumarins, furanocoumarins, phthalides, polysaccharides, benzofurans, polyacetylenes, and many others. In this context, the aforementioned chemical classes will be detailed with more insights on *Angelica dahurica*. Herein, the different isolated components were arranged according to the chemical class to which they belong.

### 3.1. Essential Oil (Table 2)

Many essential oil components had been reported from members of genus *Angelica,* especially their roots. The main reported essential oil components were α-pinene, limonene, α- and *β*-phellandrene, *p*-cymene, *β*-ocimene, *trans*-carveol, and many others. They carry vast biological activities, presented mainly by the anti-oxidant and antimicrobial activities (see Section 4. Pharmacological activities). Thus, the essential oil components isolated from genus *Angelica* can be detailed as followed.

Shchipitsyna and Efremov (2011) evaluated the chemical profile of the essential oil of *Angelica archangelica* (roots, flower heads, and seeds) from Kemerovo Oblast. The essential oil was analyzed by GC/MS, and it contained 20 major components *viz.* α-pinene, camphene, *β*-pinene, *β*-myrcene, α-phellandrene, Δ^3^-carene, *β*-phellandrene, *β*-*cis*-ocimene, *β*-transocimene, *β*-copaene, *β*-bourbonene, *β*-elemene, α-cedrene, -α-bergamotene, *β*-cedrene, *cis*-γelemene, *β*-(*Z*)-farnesene, *β*-humulene, germacrene D, bicyclogermacrene, (*E*,*E*)-α-farnesene, *γ*-cadinene, *δ*-cadinene, α-cadinene, and α-cadinol [51]. In another piece of research, the *A. dahurica* essential oil was found to be rich in *α*-pinene (46%), followed by sabinene (9%), myrcene (5%), 1-dodecanol (5%), and terpinen-4-ol (4%), while the root essential oil of *A. pubescentis* showed *α*-pinene (37%), *p*-cymene (11%), limonene (8%), and cryptone (6%) [42,52]. The *A. sinensis* essential oil held 3-N-butylphthalide, butylidene phthalide, ligustilide, and di-iso-octyl phthalate as its major ingredients [53,54].

The essential oil content of *A. sinensis* Radix was (0.4–0.7%) rich with *n*-butylidenephthalide, ligustilide, *n*-butyl-phthalide, ferulic acid, nicotinic acid, and succinic acid as the main constituents [55]. Nivinskienë et al., 2005, found that the essential oil of *A. archangelica* seed was rich in *β*-phellandrene (33–63%) and *α*-pinene (4–12%) [56]. The *A. archangelica* roots essential oil carried *α*-pinene (21%), *δ*-3-carene (16%), limonene (16%), and *α*-phellandrene (8%) as its main components [57]. Nivinskienë et al., 2005, studied the essential oil of *A. archangelica* roots collected from three regions during the period of 1995–2002. The main constituents were *α*-Pinene (15–20%) from Svencionys and Prienai, while *β*-phellandrene (13–18%) and *α*-pinene (11–15%) were the major components from Vilnius. From Lithuania, the essential oil contained (67–79%) monoterpenes, (9–19%) sesquiterpenes, and (3–6%) macrocyclic lactones [56]. Chauhan et al., 2016, found that dillapiole (35–91%) and nothoapiole (0.1–62%) were the main essential oil components isolated from the rhizomes of *A. archangelica* collected from the Western Himalayan region [39]. On the other side, Pasqua et al., 2003, studied the effect of the development stage on the essential oil formation in *A. archangelica* subsp. *archangelica* roots, where a high content of *α*- and *β*-phellandrene was found only in taproots larger 5 mm in diameter [58].

Irshad, et al., 2011, studied the essential oil content of the *A. glauca* whole plant from Jammu and Kashmir. The essential oil contained *α*-phellandrene (18%), *trans*-carveol (16%), *β*-pinene (14%), *β*-caryophyllene (8%), and *β*-caryophyllene oxide (8%) as its major compounds [59]. Similarily, Agnihotri et al., 2004, analyzed the essential oil composition of the *A.glauca* aerial parts from the Kashmir Valley, Himalaya, India. The essential oil was rich in *α*-phellandrene (13%), *trans*-carveol (12%), and *β*-pinene (11%) [40]. In another similar study, the *A. glauca* roots’ essential oil from two alpine Himalayan locations, Uttarakhand, India showed the presence of (*Z*)-ligustilide (40–53%) and (*Z*)-butylidene phthalide (20–32%) [60].

The essential oil *A. gigas*, *A. sinensis,* and *A. acutiloba* rhizomes were analyzed by a solvent-free solid injector method. The major components were coumarin derivatives *viz.* decursinol angelate (16%) and decursin (29%) succeeded by lomatin (10%), and marmesin (9%) in *A. gigas,* while the main constituents in *A. sinensis* were butylidene dihydro-phthalide, (15%), butylidene phthalide (14 %), furfural (16%), and camphene (10%), while butylidenephthalide (17%) and furfural (13%) represented the major components in *A. acutiloba* [61]. Sowndhararajan et al., 2017, detected the essential oil of the *A. gigas* root by steam distillation and supercritical carbon dioxide extract (SC-CO_2_). It was found that the essential oil mainly composed of monoterpene hydrocarbons (52%), followed by oxygenated sesquiterpenes (25%). The main monoterpenes were *α*-pinene (28%), *β*-eudesmol (14%), nonane (8%), and *γ*-eudesmol (5%); these were the major components in the essential oil of the *A. gigas* root [36]. On the other hand, Seo et al., 2007, identified decursin (40%) and decursinol angelate (28%) as the major components using SC-CO_2_, while *α*-pinene (30%) was also the main component identified from *A. gigas* using simultaneous steam distillation and extraction methods [62].

Chen et al., 2014, compared the essential oil content of *A. acutiloba* isolated from different organs (roots, stems, and leaves) through steam distillation and headspace solid-phase micro extraction (HS-SPME). In the steam distillation, *n*-butyl phthalide, *γ*-terpinene, *p*-cymene, and *cis*-*β*-ocimene represented the main components. Using HS-SPME, *γ*-terpinene and *p*-cymene were the main constituents [63]. Cavaleiro et al., 2015, found that the essential oil of the *A. major* root contained *α*-pinene (21%) and *cis*-*β*-ocimene (30%) as its major components [64].

Mohammadi et al., 2010, found that the essential oil content of the *A. urumiensis* leaves showed *α*-cadinol (20%), hexahydrofarnesyl acetone (10%), and 1-dodecanol (7%) as its major components, while *α*-cadinol (9%) and *δ*-cadenine (6%) were the main identified components from the stem essential oil of the same plant [65].

Simonović et al., 2014, identified *β*-phellandrene, *α*-pinene, and *α*-phellandrene as major components from the *A. pancicii* aerial parts’ essential oil [66]. Similarily, caryophyllene oxide (61%) and *α*-pinene (67%) were the main components from the *A. viridiflora* and *A. cincta* aerial parts’ essential oils, respectively [67].

Two hundred and thirty nine compounds were identified from the GC/MS analysis of three *Angelica* species (*A. sinensis*, *A. dahurica,* and *A. pubescens*). The main identified constituents were osthole (44.61%), obepin (0.59–86.58%), undecanol (8.58%), *α*-muurolene (7.95%), *cis*-anethol (9.11%), *E*-ligustilide (0.14–81.14%), (-)-spathulenol (0.08–1.21%), (-)-terpinen-4-ol (4.91%), 2-butylthiolane (5.76%), and *α*-bisabolol (3.80%) [68].

**Table 2:** Essential oil composition of selected species from genus *Angelica*.

*Angelica* Species	Part Used	Essential Oil Components	Reference(s)
*A. archangelica*	roots, flower heads, and seeds	*α*-pinene, camphene, *β*-pinene, *β*-myrcene, α-phellandrene, Δ^3^-carene, *β*-phellandrene, *β*-*cis*-ocimene, *β*-transocimene, *β*-copaene, *β*-bourbonene, *β*-elemene, α-cedrene, -α-bergamotene, *β*-cedrene, *cis*-γelemene, *β*-(*Z*)-farnesene, *β*-humulene, germacrene D, bicyclogermacrene, (*E*,*E*)-α-farnesene, *γ*-cadinene, *δ*-cadinene, α-cadinene, and α-cadinol	[51,56,57]
	rhizome	Dillapiole and nothoapiole	[39]
*A. archangelica* subsp. *archangelica*	root	*α*- and *β*-phellandrene	[58]
*A. dahurica*	root	*α*-pinene, sabinene, myrcene, 1-dodecanol, and terpinen-4-ol.	[42,52]
	root	*cis*-anethol, undecanol, *α*-muurolene, and (2*E*)-2-decenal	[68]
*A. pubescentis*	root	*α*-pinene, *p*-cymene, limonene and cryptone.	[42,52]
*A. sinensis*	rhizome and root	3-N-butylphthalide, butylidene phthalide, ligustilide, di-iso-octyl phthalate, ferulic acid, nicotinic acid, and succinic acid	[53,54,55]
	rhizome	Butylidene dihydro-phthalide, butylidene phthalide, furfural, and camphene	[61]
	root	*E*-ligustilide and (-)-spathulenol	[68]
*A. glauca*	whole plant	*α*-phellandrene, *trans*-carveol, *β*-pinene, *β*-caryophyllene, and *β*-caryophyllene oxide	[59]
	aerial parts	*α*-phellandrene, *trans*-carveol, and *β*-pinene	[40]
	root	(*Z*)-ligustilide and (*Z*)-butylidene phthalide	[60]
*A. gigas*	rhizome	Decursinol angelate, decursin, lomatin, and marmesin	[61]
	root	*α*-pinene, *β*-eudesmol, nonane, *γ*-eudesmol, decursin, and decursinol angelate	[36,62]
*A. acutiloba*	rhizome	Butylidenephthalide and furfural	[61]
	roots, stems, and leaves	*n*-butyl phthalide, *γ*-terpinene, *p*-cymene, and *cis*-*β*-ocimene (steam distillation) *γ*-terpinene and *p*-cymene (HS-SPME)	[63]
*A. major*	root	*α*-pinene and *cis*-*β*-ocimene	[64]
*A. urumiensis*	leaves	*α*-cadinol, hexahydrofarnesyl acetone, and 1-dodecanol	[65]
	stem	*α*-cadinol and *δ*-cadenine	[65]
*A. pancicii*	aerial parts	*β*-phellandrene, *α*-pinene, and *α*-phellandrene	[66]
*A. viridiflora* *A. cincta*	aerial parts	Caryophyllene oxide and *α*-pinene	
*A. pubescens*	root	Osthole, obepin, undecanol, *α*-muurolene, *cis*-anethol, *E*-ligustilide, (-)-spathulenol, (-)-terpinen-4-ol, 2-butylthiolane, and *α*-bisabolol	[68]

### 3.2. Coumarins and Furanocoumarins (Table 3)

**Figure 2:**
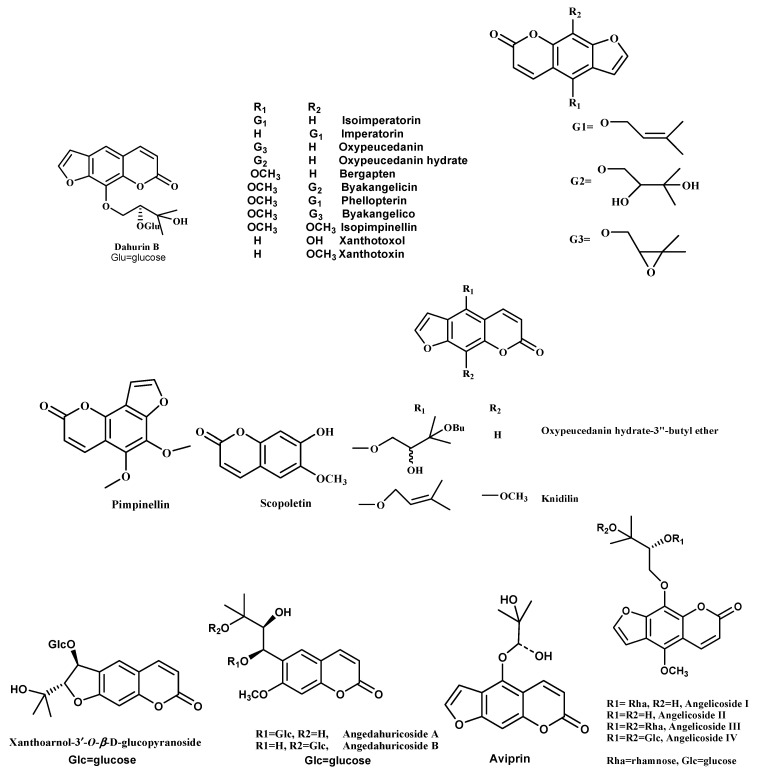
Structures of coumarins and furanocoumarins isolated from genus *Angelica*.

Earlier research on the *A. dahurica* plant showed the isolation of about 20 coumarins and 3 coumarin glycosides [69]. Zhao et al. (2007) focused on the water-soluble constituents from the fresh material of this plant. Here, the isolation and structure elucidation of the new coumarin glycoside named dahurin B were described. It was obtained as an optically active, yellowish amorphous powder. Dahurin B (C_22_H_26_O_11_) (Figure 2) was determined on the basis of its ESI-MS (489 [M+Na]^+^), and confirmed by ^1^H NMR and ^13^C NMR data. Detailed analysis of its ^1^H NMR, ^13^C NMR, COSY, HSQC, and HMBC spectra indicated the presence of a linear furanocoumarin glucoside and a 2-methylbutane structural unit [70].

Li and Wu (2017) investigated the chemical constituents of *A. dahurica*. Fifteen compounds have been identified as isoimperatorin (1), imperatorin (2), oxypeucedanin (3), oxypeucedanin hydrate (4), bergapten (5), byakangelicin (6), phellopterin (7), byakangelicol (8), isopimpinellin (9), xanthotoxol (10), xanthotoxin (11), pimpinellin (12), scopoletin (13) (Figure 2), *β*-sitosterol (14), and daucosterol (15) [71,72].

Kim et al. (2002) found that the *A. dahurica* roots’ methanol extract fractionation led to the isolation of three furanocoumarins, namely isoimperatorin, imperatorin, and oxypeucedanin (Figure 2). The isolated compounds inhibited AChE activity in a dose-dependent manner (IC_50_ 63.7 to 89.1 µM) [73]. In another study, five furanocoumarins (isoimperatorin, oxypeucedanin hydrate-3”-butyl ether, imperatorin, knidilin, and oxypeucedanin hydrate) (Figure 2) were isolated from the root of *A. dahurica* by repeated silica-gel column chromatography [74].

Yang et al. (2020) utilized a quantitative ^1^HNMR method (^1^H-qNMR) for the determination of the imperatorin, akangelicin, and oxypeucedanin content in *A. dahurica,* which are part of traditional Chinese medicine (TCM). The extraction was performed using an ultrasonication-assisted extraction method. The quantitative proton NMR measurements were executed on a 600-MHz spectrometer with hydroquinone as the internal standard reference in a deuterated dimethyl sulfoxide (DMSO-d6) solvent. Quantification was carried out using the ^1^H resonance signals for hydroquinone (6.55 ppm) and for imperatorin, byakangelicin, and oxypeucedanin, (7.68, 7.38–7.39 and 6.38–6.39 ppm), respectively. The linearity, limit of quantitation, precision, reproducibility, stability, limit of detection, and methodology were evaluated, and the results were good. The newly developed method has been applied to determine the three coumarins in *A. dahurica* [75].

Zhao et al. (2018) isolated ten compounds from the roots of *A.* dahurica, which were namely xanthoarnol-3′-*O*-*β*-D-glucopyranoside, angedahuricoside A, angedahuricoside B (Figure 2), isofraxidin-7-*O*-*β*-D-glucopyranoside, fraxidin-8-*O*-*β*-D-glucopyranoside, (-)-marmesinin, (2′S,3′R)-3′-hydroxymarmesinin, hyuganoside V, daucosterol, and sucrose [76]. In another study, six furocoumarins were isolated from the methanol extract of A. dahurica Benth. The isolated compounds were imperatorin, isoimperatorin, (±)-byakangelicol, (+)-oxypeucedanin, (+)-byakangelicin, and (+)-aviprin. [77].

Shua et al. (2020) investigated the phytochemicals present in *A. dahurica* roots using different chromatographic techniques *viz.* UV, IR, NMR, and HR-ESI-MS, together with acid hydrolysis and enzymatic hydrolysis. Four furanocoumarins were isolated, namely angelicosides I–III and angelicoside IV (Figure 2). [78].

Matsuo et al. (2020) performed a phytochemical investigation of the root of *A. dahurica* using 2D NMR data, hydrolysis, and different solvent solubility, followed by either physicochemical and spectroscopic data or X-ray crystallographic analysis. The investigation resulted in the isolation of a combination of benzofuran and coumarin derivatives. [79].

Isoimperatorin is a novel compound isolated from *A. dahurica* with wide biological applications [80]. Psoralen, isopsoralen, imperatorin, isoimperatorin, phellopterin, and cnidilin represent new furanocoumarins isolated from the root of *A. dahurica* [81]. Similarly, xanthotoxol was isolated from *A. dahurica,* and it carried potential anticancer activity [82]. Through NMR, IR, and LC/MS analysis, the structure of a new coumarin named angedahurin A was determined. This coumarin was isolated from the *A. dahurica* roots [83,84,85].

**Table 3:** Coumarins and furanocoumarins isolated from *Angelica dahurica*.

No.	Compound Name	Reference(s)
1	Dahurin B	[70]
2	Isoimperatorin	[71,73,74,77,80,81]
3	Imperatorin	[71,73,75,77,81]
4	Oxypeucedanin	[71,73,75,77]
5	Oxypeucedanin hydrate	[71,74]
6	Bergapten	[71]
7	Byakangelicin	[71,77]
8	Phellopterin	[71]
9	Byakangelicol	[71,77]
10	Isopimpinellin	[71]
11	Xanthotoxol	[71,82]
12	Xanthotoxin	[71]
13	Pimpinellin	[71]
14	Scopoletin	[71]
15	Oxypeucedanin hydrate-3”-butyl ether	[74]
16	Knidilin	[74]
17	Akangelicin	[75]
18	Xanthoarnol-3′-*O*-*β*-D-glucopyranoside	[76]
19	Angedahuricoside A	[76]
20	Angedahuricoside B	[76]
21	Isofraxidin-7-*O*-*β*-D-glucopyranoside	[76]
22	Fraxidin-8-*O*-*β*-D-glucopyranoside	[76]
23	(-)-Marmesinin	[76]
24	(2′*S*,3′*R*)-3′-Hydroxymarmesinin	[76]
25	Hyuganoside V	[76]
26	(+)-Aviprin	[77]
27	Angelicosides I–IV	[78]
28	Psoralen	[81]
29	Isopsoralen	[81]
30	Phellopterin	[81]
31	Cnidilin	[81]
32	Angedahurin A	[83]

### 3.3. Phthalides

Zhang et al. (2013) evaluated the chemical and biological profiles of different *Angelica* roots collected from different geographical origins. The roots of *A. sinensis* presented ferulic acid, *Z*-ligustilide, and senkyunolide A, while butylphthalide and *Z*-butylenephthalide were isolated from *A. gigas* roots. [86]. Chao and Lin (2011) isolated about 70 compounds from *A. sinensis* (Oliv.) Diels roots. Ferulic acid represented the major component, followed by butylidenephthalide and other polysaccharides. [87]. Novel phthalide derivatives, namely oxaspiroangelioic acids A, B, and C, were isolated from the root extract of *A. sinensis* [88,89] (Table 4).

### 3.4. Polysaccharides

A glucoarabinan polysaccharide was isolated from *A. dahurica,* formed mainly of arabinose and traces of glucose. It was named (ADP80-2), and its monomeric structure is composed of →5)-α-L-Araf-(1→, →3, 5)-α-L-Araf-(1→, →6)-α-D-Glcp-(1→, with a terminal branch α-L-Araf-(1 →residue [90]. Similarly, another polysaccharide was isolated from *A. dahurica,* with its monosaccharide content formed of D-mannose, D-glucose, D-galactose, and L-arabinose, and its first isomer was formed of D-mannose, D-glucose, D-galactose, and L-arabinose, while its second isomer was composed of D-mannose, D-galacturonic acid, D-glucose, D-galactose, and L-arabinose, and the third isomer was mainly constituted by D-mannose, L-rhamnose, D-galacturonic acid, D-galactoe, and L-arabinose [91]. A new acidic polysaccharide was isolated from *A. dahurica* with a sugar and uronic acid content of 91.04% and 12.69%. This polysaccharide was composed of rhamnose, arabinose, galactose, glucose, mannose, glucuronic acid, and galacturonicacid, with the following ratios (0.09: 0.61: 1.88: 1: 0.14: 0.63: 0.03). Its chemical skeleton showed the presence of this bonding manner: →3)-Manp-(1→, →4, 6)-Galp-(1→, →4)-Galp-(1→, →3)-Glcp-(1→, →5)-Araf-(1→, →2)-Galp-(1→ (0.32:0.57:0.29:0.95:0.71:0.26 molar ratios) [92].

### 3.5. Benzofurans

Bezofurans comprise an important class of phytoconstituents belonging to the heterocyclic compounds. From *A. dahurica* roots, six benzofurans were isolated including 3-[6,7-furano-9-hydroxy4-(2″,3″-dihydroxy-3″-methylbutyloxy)]-phenyl propionic acid, 3-[6,7-furano-9-(β-D-glucopyranosyloxy)-4-(2″,3″-dihydroxy-3″- methy butyloxy)]-phenyl propionic acid, 3-[6,7-furano-9-(β-D-glucopyranosyloxy)-4-(2″,3″-dihydroxy-3″-methylbutyloxy)]-phenyl propionic acid methyl ester, cnidioside A, methylcnidioside A, and methylpicraquassioside [79].

### 3.6. Polyacetylenes

Only two polyacetylenes were reported from genus *Angelica,* namely falcarindiol and octadeca-1,9-dien-4,6-diyn-3,8,18-triol [93].

## 4. Pharmacological Activities

### 4.1. Analgesic and Anti-Inflammatory Activity

An isolate from *A. dahurica* named byakangelicol was tested against IL-1-induced COX-2 expression and PGE_2_ release in the human pulmonary epithelial cell line (A549). The isolated compound decreased the expression of IL-1 and PGE_2_ release at a dose of (10–50 M) in a dose dependent manner. Byakangelicol when used at a dose of up to 200 M showed no effect on the expression of the COX-1 enzyme. The compound also showed no effect on IL-1-induced p44/42 mitogen-activated protein kinase (MAPK) activation. Treatment of cells with byakangelicol (50 M) or pyrrolidine dithiocarbamate (PDTC; 50 M) partially inhibited the IL-1-induced degradation of I B- in the cytosol, translocation of p 65NF- B from the cytosol to the nucleus, and the NF- B-specific DNA–protein complex formation. The mechanism of inhibition by which byakangelicol could be explained was by suppression of NFB activity [94].

Choi et al., 2008, evaluated the analgesic and anti-inflammatory activities of *A. dahurica,* using acetic acid and carrageenan to induce pain and edema in rats, respectively. In the analgesic model, the rats were injected with acetic acid, and visceral pain was tested through the writhing reflex. In the inflammation model, rats were injected with carrageenan in their paws, and the volume of edema was measured. Groups treated with *A. dahurica* showed a lower writhing reflex in a dose-dependent manner, while in the inflammation model, the paw edema was reduced significantly by treatment with the extract [95].

In this study, 5-Methoxy-8-(2-hydroxy-3-buthoxy-3-methylbutyloxy)-psoralen (MP) isolated from *A. dahurica* acted as an inhibitor of the COX-2-dependent phase of prostaglandin D_2_ (PGD_2_) generation in bone-marrow-derived mast cells (IC_50_ 23.5 µM). This was further confirmed through a Western blot with specific anti-COX-2 antibodies, which caused a reduction in the production of PGD_2_, and also, in COX-2 protein levels. Moreover, MP further inhibited the production of leukotriene C_4_ (IC_50_ 2.5 µM, depending on dose). Moreover, MP helps in the degranulation reaction inhibition (IC_50_ 4.1 µM) [96].

Lee et al. (2011) descried the effects of the ethanol extract of *A. dahurica* on airway inflammation in an ovalbumin-induced airway inflammation model in mice. The group of mice treated with the extract showed significant lower airway eosinophilia, cytokine levels, including IL-4, IL-5, and TNF-*α* levels, mucus production, and IgE compared with OVA-induced mice. The extract acts by reducing airway inflammation and decreasing oxidative radical levels through the activation of heme oxygenase (HO)-1; thus the ethanol extract of *A. dahurica* can act as an allergic inflammatory modulator for asthmatic patients [97].

The essential oil of *A. dahurica* (dose 100 mg/kg) had an anti-inflammatory activity against xylene-induced ear swelling and carrageenan-induced paw edema in a mice model. The essential oil also significantly alleviated Freund’s complete adjuvant-induced arthritis in rats by improving hind-paw swelling and reducing the serum levels of nitric oxide, TNF-α, prostaglandin E_2_, and serum nitric oxide synthase activity [41]. Zhang et al. (2015) studied the relation between the GC/MS-identified metabolomics of the *A. sinensis* essential oil and their activity in rats with acute inflammation. In the carrageenan-injected rats, treatment with the essential oil of *A. sinensis* significantly rectified the levels of PGE_2_, histamine, and 5-hydroxytryptamine in the inflammatory fluid [98].

In another study, the essential oil of *A. sinensis* was tested against inflammation in carrageenan-induced acute inflammation in rats. Different processed essential oil samples were prepared for the study including: stir-fried *A. sinensis*, fried *A. sinensis* with alcohol, cooked *A. sinensis* with soil, and fried *A. sinensis* with sesame oil. All of the essential oil samples significantly inhibited PGE_2_, histamine, 5-hydroxytryptamine, and TNF-*α* release [99]. Similarly, the essential oil from *A. sinensis* showed an anti-inflammatory activity against the lipopolysaccharide (LPS)-induced inflammation in rats. The mechanism behind such activity is due to the regulation of the Krebs cycle, improving the glucose content, and thus restoring the fatty acid metabolism [100]. Li et al. (2016) also analyzed the anti-inflammatory activity of the *A. sinensis* essential oil on the LPS-induced acute inflammation in rats. The essential-oil-treated groups had a decrease in the levels of the inflammatory markers *viz.* cytokines (TNF-α, IL-1*β*, and IL-6), mediators (histamine, 5-hydroxytryptamine, PGE_2_, and nitric oxide), enzymes (nitric oxide synthase and cyclooxygenase 2), thus inducing anti-inflammatory and hepatoprotective activities compared to the control groups [101].

In this current study, *Angelica* polysaccharide (AP) was tested as an anti-inflammatory agent against mast cells and their molecular mechanism. AP was tested at doses of (50, 100, and 200 μg/mL), and the polysaccharide significantly reduced histamine, *β*-hexosaminidase, leukotrienes C4 (LTC4), IL-1, IL-4, TNF-*α*, IL-6, and human monocyte chemotactic protein-1 release; in addition, it inhibited Ca^2+^ entry to mast cells. Moreover, it downregulated the protein expressions of p-Fyn, p-Akt, p-P38, IL-4, TNF-*α*, and NF-*κ*B p65 [102].

Li and Wu (2017) reported the isolation of fifteen compounds from *A. dahurica*. The isolated compounds inhibited the secretion of inflammatory mediators such as TNF-*α*, IL-1*β*, and IL-4. The anti-inflammatory activity was related to their inhibitory activity against the expression of the cytokine-producing genes, and, thus, the inhibition of nuclear factor-κB activation [71].

Isoimperatorin, isolated from *A. dahurica* roots, possessed potent anti-inflammatory activity in vitro. The anti-inflammatory activity was assayed through an MTT assay, real-time PCR, ELISA, and western blot, together with molecular docking, to assess the binding of isoimperatorin and myeloid differentiation protein-2 (MD-2), and elucidate the possible anti-inflammatory mechanism. Isoimperatorin significantly inhibited the release of NO, TNF-*α*, IL-6, and IL-1*β* usually secreted in inflammation. Real-time PCR resulted in the observation that isoimperatorin reduced the mRNA expressions of iNOs, COX-2, TNF-*α*, IL-6, and IL-1*β*. Isoimperatorin inhibited the formation of the proteins associated with the LPS-TLR4/MD-2-NF-κB signaling pathway, as shown in the Western blot. In addition to that, molecular docking showed the binding between isoimperatorin and MD-2 [103].

A traditional Chinese medicine named Huoxiangzhengqi oral liquid (HXZQ-OL) and is constituted of *A. dahurica* was evaluated for its potential anti-inflammatory and anti-allergic activity. The anti-allergic activity was tested using IgE/Ag-mediated RBL-2H3 cells with doses of (43.97, 439.7, and 4397 μg/mL) in vitro. The release of cytokines and eicosanoids were quantified using ELISA. RT-qPCR was utilized to assess cytokine gene expression. Immunoblotting analysis showed the mechanism of action of this traditional formula. The formula was also tested in vivo in mice through the passive cutaneous anaphylaxis (PCA) assay, where mice were orally administrated with the formula using doses of (263.8, 527.6, and 1055 mg/kg/d) for seven consecutive days. The formula successfully inhibited mast cell degranulation with an IC_50_ value of 123 μg/mL, and also prevented both the generation and secretion of IL-4 (IC_50_ 171.4 μg/mL), TNF-*α* (IC_50_ 88.4 μg/mL), LTC4 (IC_50_ 52.9 μg/mL), and PGD_2_ (IC_50_ 195.8 μg/mL). The formula also inhibited IL-4 and TNF-*α* mRNA expression. Moreover, the formula helped in the attenuation of the IgE-mediated PCA, with a 55% suppression of Evans blue exudation in mice (527.5 mg/kg) [104].

The essential oils isolated from different *Angelica* species, namely *A. sinensis*, *A. dahurica,* and *A. pubescens,* were evaluated for their anti-inflammatory activity using an ear edema model caused by 12-*O*-tetracycline-propylphenol-13-acetic acid (TPA) in mice, compared to Ibuprofen. Levels of the inflammatory markers and mediators such as TNF-*α*, COX-2, IL-6, and RelA (p65) were recorded by the immune-histochemical method. The tested essential oils significantly decreased the levels of TNF-*α*, COX-2, IL-6, and p65 [68].

Three polysaccharide isomers were purified from *A. dahurica,* and they showed scavenging activity against both DPPH free radical and hydroxyl free radical, along with ferric reducing power, which explains their potential use as natural antioxidants [91].

The *A. dahurica* extracts’ anti-inflammatory activity was evaluated using a complete Freund’s adjuvant-induced inflammatory pain mice model. The extract successfully reduced the mechanical and thermal hypersensitivities in a CFA-induced inflammatory pain model in mice; thus, the extract could play a role in chronic inflammation management [105].

The anti-inflammatory potential of the furanocoumarins (imperatorin and byakangelicin) isolated from the *A. dahurica* root extract was analyzed in order to pinpoint which furanocoumarin induces such activity in hepatocytes. The methanol root extract was fractionated using ethyl acetate, butanol, and water. Rat hepatocytes were grouped into two categories: the first treated with I)-1*β*, the second with each fraction (ethyl acetate, butanol, and aqueous fractions) for 8 h. Levels of both NO production and lactate dehydrogenase were recorded. The ethyl acetate fraction markedly suppressed NO production without showing cytotoxicity and decreased iNOS expression in hepatocytes. Five furanocoumarins were isolated from the ethyl acetate fraction, namely isoimperatorin, imperatorin, phellopterin, oxypeucedanin, and oxypeucedanin methanolate. Phellopterin and oxypeucedanin methanolate significantly suppressed NO production and reduced the mRNA expression of iNOs and TNF-*α*. A comparison of their chemical structures suggests that a methoxy group at carbon 5 and a side chain at carbon 8 in the furanocoumarin skeleton may be essential for NO production suppression. [106].

### 4.2. Cytotoxic Activity

Lee et al. (2020) utilized the chloroform fraction of the roots of *A. dahurica* to isolate eight furanocoumarins. Psoralen, xanthotoxin, and bergapten showed potent inhibitory activity against IR-induced migration at a non-cytotoxic concentration (50 μM) in human NSCLC A549 cells, and thus played an important role in cancer metastasis treatment [107].

The anti-cancer activity of the *A. dahurica* extract against the HT-29 colon cancer cell line was evaluated. The measured parameters included: cell viability, apoptotic, and necrotic activities. The non-polar extract of *A. dahurica* significantly reduced the gene expression of p53, Bcl, and Bax, and enhanced apoptosis through caspase cascade and cell cycle arrest. The polar fractions (ethanol-ethyl acetate) had cytotoxic activity in HT-29 cancer cells. Imperatorin and isoimperatorin were the main causes of such cytotoxic activity [108]. Isoimperatorin, isolated from *A. dahurica*, had a pronounced activity on the signaling pathway involved in cancer cell metastasis in colon and hepatic cancers when compared to its isomer, imperatorin [80]. A novel compound isolated from *A. dahurica* called xanthotoxol showed promising cytotoxic activity against non-small cell lung cancer development via the inhibition of cell viability, colony formation capacity, DNA replication, cell cycle transition, migration and invasion, and induction of apoptosis in NSCLC cells. It also suppresses NSCLC xenograft growth in vivo without toxicity [82].

A new acidic polysaccharide was isolated from *A. dahurica,* which showed significant cytotoxicity in an in vivo model of tumors in mice *via* enhancing the activities of spleen lymphocytes and natural killer (NK) cells and increasing the levels of IL-2 and TNF-*α*. Tumor cell apoptosis ranged from 7.54% to 19.32% (dose of 100 and 200 mg/kg) and was confirmed by pathological samples [92].

A new coumarin named angedahurin A was isolated from *A. dahurica* roots. Its potential cytotoxic activity against MG-63 human osteosarcoma cell lines was evaluated, where it showed marked cytotoxicity with IC_50_ 7.2 μM, compared to 5-florouracil (IC_50_ 32.4 μM) [83].

Cholecystokinin octapeptide was used to screen the cytotoxicity of alloimperatorin on HeLa, SiHa, and MS-751 cancer cell lines, and flow cytometry was used to record apoptosis. Apoptosis was confirmed through mitochondrial membrane potential, Western blot, and fluorescent PCR. Alloimperatorin showed potent cytotoxic activity against Hela cells (IC_50_ 116.9 μM), by accelerating the apoptotic rate of HeLa cells and decreasing its mitochondrial membrane potential. Alloimperatorin also enhanced the expression of caspases 3, 8, and 9 in the Western blot [109].

### 4.3. Anti-Oxidant Activity

Pervin et al. (2014) evaluated the anti-oxidant activity of the aqueous and ethanol extracts of *A. dahurica* root. Antioxidant activity was tested using DPPH, ABTS assays, hydroxide radical scavenging activity, and lipid peroxidation (dose 0.12–2.0 mg/mL). The fractions scavenged the DPPH and ABTS radicals (IC_50_ 0.32 and 0.20 mg/mL), respectively, for the aqueous extract, and (0.24 and 0.13 mg/mL), respectively, for the ethanol extract. The two extracts had potent reducing power and inhibited superoxide dismutase, catalase and DNA damage. It also inhibited the production of NO in a dose-dependent manner in lipopolysaccharide-treated RAW264.7 cells [110].

### 4.4. Antimicrobial Activity

Yang et al., (2020) evaluated the antibacterial activity of *A. dahurica*. Thirty Sprague–Dawley rats were divided into three groups: normal saline (NS), extract-treated, and biomycin-ointment-treated (BO). *S. aureus* was used to induce infection in dorsal excisions made on each rat, and each group received treatment once daily for 7 days. Treatment with the extract led to a smaller wound area compared to the two other groups, and the total bacterial count was lower as well. Body temperature and inflammatory markers *viz.* TNF-*α* and IL-6 levels markedly decreased in the extract-treated group. In wound tissue samples, the extract-treated group showed faster scab formation, denser granulation tissue, thicker epidermis, and more angiogenesis markers than the other groups [111,112].

The essential oil isolated from the Korean *Angelica,* especially sabinene and *m*-cresol, had strong antifungal activity against different species of *Aspergillus* and *Trichophyton* (MIC 125–1000 μg/mL). Moreover, the essential oil showed synergistic activity with itraconazole [113].

Irshad et al., 2011, tested the antimicrobial activity of the *A. glauca* essential oil against different bacterial (*Staph. aureus*, *B. subtilis*, *E. coli,* and *Past. multocida*) and fungal (*C. albicans*, *M. canis*, *A. flavus,* and *F. solani*) strains. The extract was active against *E. coli* and *Staph. aureus* (MIC 141.3 and 159.3 µg/mL), respectively, and *M. canis* (MIC178.1 µg/mL) [59].

Fraternale et al., 2014 & 2016, proved that the essential oil of the *A. archangelica* root had antimicrobial activity against *C. difficile*, *C. perfringens*, *Ent. faecalis*, *E. limosum*, *P. anaerobius,* and *C. albicans*. Moreover, the essential oil showed weaker activity against bifido bacteria and lactobacilli. In another study, the same species’ essential oil showed antifungal activity against some species of the Fusarium genus, *B. cinerea* and *A. solani* [57,114]. A combination of equal proportions of the *A. archangelica* essential oil, phenyl ethyl alcohol, and *α*-terpineol inhibited *A. flavus* NKDW-7 (afla toxigenic strain) and afla toxin B1 production (2.25 and 2.0 μL/mL), respectively [115].

Cavaleiro et al., 2015, tested the *A. major* essential oil (*α*-pinene and *cis*-*β*-ocimene) against different strains of yeasts and molds. The essential oil showed a broad spectrum of activity against all tested fungi (animal and human pathogenic species or spoilage fungi) *viz. Candida spp.*, *C. neoformans*, *Aspergillus spp.* and dermatophytes. *α*-pinene was more potent compared to *cis*-*β*-ocimene [64]. Similarily, *A. sinensis* and *A. dahurica* essential oils had significant antibacterial activity against mastitis-causing pathogens (*Staph. aureus*, *Staph. Chromogenes,* and *S. uberis*) [116]. In addition to that, the essential oil of *A. pubescentis* exhibited weak antifungal activity against *Colletotrichum acutatum*, *C. fragariae,* and *C. gloeosporioides*. On the other hand, the *A.dahurica* root essential oil showed no activity against the same tested fungal strains [42].

In a recent study, the gold and copper nanoparticles prepared from the leaf extract of *A. keiski* were evaluated for their antibacterial activity, where they showed interaction with the bacterial cell wall of some tested Gram-negative bacteria, leading to cell wall rupture. The copper nanoparticles were more effective as antibacterial agents, showing wider zones of inhibition against *E. coli*, *S. typhimurium,* and *Staph. aureus* [117].

*A. dahurica* inhibited the ability of *P. aeruginosa* to form biofilm, thus reducing its resistant power and limiting its growth. In this context, coumarins such as imperatorin and isoimperatorin isolated from the extract of *A. dahurica,* combined with antibiotics such as ampicillin and ceftazidime, were evaluated against *P. aeruginosa*. The combination treatment showed superior antibacterial activity and reduced the biofilm formation in *P. aeruginosa* [118]. Four furanocoumarins were isolated through bioactivity-guided isolation from the 70% ethanol extract of *A. dahurica* roots. The isolated compounds were evaluated against the influenza virus. They acted by inhibiting cytopathic effects, which acted as a marker for their antiviral activity against the Chik influenza A (H_1_N_1_) and swine flu (H_9_N_2_) viruses. The most active compound was subjected to in-depth mechanistic studies *viz.* viral protein synthesis inhibition, cytopathic inhibition in different phases of the viral replication cycle, neuraminidase (NA) inhibition, antiapoptotic activity using flow cytometry, and immunofluorescence. The active compound showed anti-influenza-A activity through the inhibition of the early phase of the viral replication cycle, and not *via* direct neutralization of surface proteins, such as hemagglutinin and NA, and abnormal apoptosis [119].

### 4.5. Effects on Cardio- and Cerebrovascular Systems

Lee et al. (2015) studied the pharmacological mechanism behind the anti-hypertensive effect of *A. dahurica,* leading to its vasorelaxant effect. The 70% methanol extract of *A. dahurica* root was evaluated on the vasorelaxation of the rat thoracic aorta. Isolated rat aortic rings were suspended in organ chambers containing 10 mL Krebs–Henseleit (K–H) solution, and placed between two tungsten stirrups and connected to an isometric force transducer. Differences in tension were documented through isometric transducers connected to a data acquisition system. The extract had a dose-dependent relaxation in both the endothelium-intact and endothelium-denuded aortic rings precontracted with phenylephrine (PE; 1 μM) or potassium (KCl; 60 mM) in the K–H solution. Pre-treatment of the rings with the extract (1 mg/mL) successfully inhibited the Ca-induced vasocontraction of the aortic rings. Thus, the *Angelica* extract had a vasorelaxant effect, and this effect was mediated through an endothelium-independent pathway, which involves extracellular calcium influx inhibition *via* the receptor-operated Ca^2+^ channel and voltage-dependent calcium channel pathways [30].

The pharmacological activities of the extracts from *A. sinensis* and its active compounds on cardio- and cerebrovascular systems have been introduced in Modern Research and Application of Chinese Medicinal Plants, published in 2000 [120]. The water extract of the roots of *A. sinensis* and ferulic acid (FA) were able to inhibit rat platelet aggregation induced by adenosine diphosphate (ADP) and collagen in vitro. At the dose of 0.4–0.6 mg/kg (iv), sodium ferulate inhibited aggregation induced by ADP and collagen in rats [121]. Neither platelet aggregation nor arterial PGI_2_-like substance release was observed following the IV administration of sodium ferulate at a dose of 300 mg/kg. In a combined sodium ferulate and acetylsalicylic acid regimen, platelet aggregation and production of the TXA_2_-like substance were inhibited by 65% and 84%, respectively, while the production of the arterial PGI_2_-like substance remained unchanged. The combined treatment using sodium ferulate and acetylsalicylic acid could potentiate the antiplatelet action without inhibiting arterial PGI_2_-like substance release, suggesting that they may be valuable for the treatment of thromboembolic diseases [122].

Coumarin and its derivatives, natural anti-coagulants in *Angelica spp.*, have been associated with both the bioactivity and toxicity of the plants, although *A. sinensis* contains a lower coumarin content compared to other closely related species [123]. FA, one of the constituents of *A. sinensis*, could inhibit the polymerization of platelets in blood circulation. It retards platelet release of 5-hydroxy-tryptamine (5-HT) and ADP [55]. Both FA and an aqueous extract of *A. sinensis* were found to inhibit platelet aggregation and serotonin release [55]. Due to the untold number of constituents, several pharmacological actions might be attributed to *A. sinensis*. Such characteristics include anticoagulation and antiplatelet activities [55], as well as hematopoiesis [124].

In an in vitro study of the preovulation follicles on chicken blood vessels, the extract of *A. sinensis* potentiated angiogenic activity, by enhancing the endothelial blood vessel growth through promoting the phosphorylation reaction involved in the activation of the endothelial growth factor receptor II [125].

### 4.6. Neuroprotective Action

LIG has been shown to reduce ischemic brain injury *via* anti-apoptotic pathways in a study by Chen et al., 2011, who investigated the neuroprotective potential of LIG after experimental subarachnoid hemorrhage (SAH) in rats. Rats with SAH, induced using the established double hemorrhage model, were studied with and without LIG treatment. Mortality, neurobehavioral evaluation, brain water content, blood–brain barrier (BBB) permeability, and vasospasm assessment of the basilar artery were measured on days 3 and 7 after injury. Additional testing was done to evaluate apoptosis using TdT-mediated dUTP-biotin nick end-labeling staining, as well as immunohistochemistry and Western blotting, to identify key proapoptotic/survival proteins such as p53, Bax, Bcl-2, and cleaved caspase-3. LIG treatment reduced mortality, neurobehavioral deficits, brain edema, BBB permeability, and cerebral vasospasm. In addition, treatment reduced the number of apoptotic cells in the surrounding brain injury site, which accompanied a marked downregulation of proapoptotic proteins, p53, and cleaved caspase-3 [126,127].

The extract of *A. sinensis* was orally administered to different groups of rats (0.5–1 g/Kg) to test its neuroprotective activity. The tested extract showed upregulation of several proteins in the hippocampus that are directly linked to the neuronal survival in this area, giving hope for treatment in cerebral ischemia patients [128]. Different *Angelica sinensis* extracts were evaluated against chronic unpredictable mild stress (CUMS)-induced depression in rats. A CUMS-inducing procedure was performed in male rats to induce depression. They were exposed to it for 5 weeks, which led to depressive behaviors in rats, involving a reduction in sucrose consumption and reduced sucrose preference ratios in a sucrose preference test, lengthened immobility times, lesser struggling time in a forced-swim test, and decreased locomotor activity in an open field test. Moreover, the expression of brain-derived neurotrophic factor (BDNF), and the phosphorylation of cAMP-response element binding protein and extracellular signal-regulated protein kinase (ERK 1/2) were significantly decreased in the hippocampus in depressed rats. Treating the depressed rats with *Angelica sinensis* extracts (1 g/kg) normalized their depression-related behaviors and molecular profiles [129].

### 4.7. Immune Support and Hematopoiesis

FA-induced anti-immobility was prevented by pretreatment with PCPA, WAY-100635, ketanserin, sulpiride, SCH233390, haloperidol, and yohimbine, independently. CRH, ACTH, and 5-HT were significantly decreased, but ghrelin was apparently increased compared with vehicle. In summary, FA induced anti-depression and prokinetics *via* inhibiting 5-HT, norepinephrine and dopamine reuptakes, regulating HPA axis, increasing ghrelin, and stimulating jejunal contraction simultaneously [130]. Lymphocyte proliferation assays indicate that *A. sinensis* consistently exerts an immune-stimulatory effect [131,132]. A high-molecular-weight polysaccharide found in *A. sinensis* has demonstrated immune-stimulating activity and a blood-tonifying effect by inducing hematopoiesis in the bone marrow. This is accomplished, in part, by either the direct or indirect stimulation of macrophages, fibro-blasts, erythrocytes, granulocytes, and lymphocytes, and could induce an increased secretion of human growth factors from muscle tissue. The evidence of hematopoiesis is further supported by the presence of significant amounts of vitamin B12, folinic acid, and biotin in *A. sinensis* [124].

### 4.8. Antifibrotic Action

A mixture of *Angelica* and *Astragalus* demonstrated antifibrotic activity in a recent animal study. Rat models with chronic puromycin-induced nephrosis were treated with either an *Angelica* and *Astragalus* mixture (3 mL/d), or Enalapril (10 mg/kg). The normal control group received saline, and another group received puromycin with no treatment [133]. After 12 weeks, the untreated rats showed marked renal fibrosis. However, the *Angelica* and *Astragalus* mixture significantly retarded the progression of renal fibrosis and deterioration of renal histological damage, with effects comparable to Enalapril [133].

Geng et al., 2017, found that the ethanol and aqueous extracts from either ASR or AR led to a reduction in the area of collagen fibers and the extent of alveolus inflammation, and also the content of Hyp in lung tissue and lung index. The above-mentioned extracts may protect against rat pulmonary fibrosis induced by bleomycin and showed a protective effect on pulmonary fibrosis in rats in the early period [134].

### 4.9. Antispasmodic Activity

LIG, butylidenephthalide, and butylphthalide were found to have antispasmodic activity against rat uterine contractions and in other smooth muscle systems. The components were characterized as non-specific anti-spasmodic, with a mechanism different from papaverine [123,135].

### 4.10. Hepatoprotective Activity

Cao et al. (2018) investigated the hepatoprotective effects of *Angelica sinensis* polysaccharide (ASP), an active constituent derived from the aqueous extract of *Angelica sinensis*, in rats exposed to an APAP overdose. The mechanisms underlying the activity of this compound were also considered. Specifically, serum and hepatic biochemical parameters including alanine aminotransferase (ALT), aspartate transaminase (AST), glutathione (GSH), malondialdehyde (MDA), and superoxide dismutase (SOD) were evaluated, and key proteins involved in hepatic apoptosis, including cleaved caspase-3, Bax, and Bcl-2 were quantified. In vivo, H&E staining reveals that ASP reduces the degeneration of hepatocytes and the amount of cytoplasmic vacuolation in rats exposed to an overdose of APAP. ASP markedly alleviated liver injury *via* an increase in GSH levels and the inhibition of hepatic apoptosis. In vitro, ASP significantly elevated the survival rate of rat primary hepatocytes exposed to an overdose of APAP. The beneficial effect might be, at least in part, due to the amelioration of lipid peroxidation and oxidative stress, along with the inhibition of apoptosis. Taken together, their findings reveal that ASP has the potential to be used as a hepatoprotective agent for the management of APAP-induced liver injury [136].

### 4.11. Antidiabetic

Park et al. (2016) found that phellopterin, isolated from *Angelica dahurica*, can act as a powerful anti-diabetic agent. This compound can elevate insulin secretion and enhance glucose tolerance in vivo *via* the activation of GPR119. Mice treated with this *Angelica* extract showed an enhanced glucose tolerance and increased insulin secretion, while giving them repeated doses of the *Angelica* extract or the *n*-hexane fraction led to an improvement in the glucose tolerance in diabetic mice. Imperatorin, phellopterin, and isoimperatorin were isolated from the active fraction of the extract from which phellopterin caused the activation of GPR119 and GLP-1, increased insulin secretion in vitro, and enhanced glucose tolerance in normal and diabetic mice. Thus, this pure compound could play an important role in type II diabetes treatment [137,138,139].

### 4.12. Skin Permeation Enhancer

Essential oils, being lipid soluble, could help in overcoming the skin permeation of drugs through bypassing the *stratum corneum* barrier. In this context, [140] evaluated the potential activity of five essential oil samples (clove, *Angelica*, *Chuanxiong*, *Cyperus,* and cinnamon) as permeation enhancers to help in drug delivery through the transdermal route. Ibuprofen was selected and applied using dysmenorrheal model mice. *Chuanxiong* and *Angelica* essential oils effectively enhanced the transdermal drug delivery of ibuprofen.

Similarly, Jiang et al. (2017) studied different essential oils *viz.* turpentine, *Angelica*, *Chuanxiong*, *Cyperus*, cinnamon, and clove oils (concentration 3% *w*/*v*) as skin permeation enhancers for ibuprofen in rats. The studied essential oil samples caused a significant increase in skin penetration by the drug effect, with less skin irritation [141].

### 4.13. Estrogenic Activity

Piao et al. (2006) isolated eleven furanocoumarins as new and effective phytoestrogens from *A. dahurica*. They were effective in treating menopausal symptoms, showing estrogenic activity on the Ishikawa cell line. 9-hydroxy-4-methoxypsoralen and alloisoim-peratofin significantly induced alkaline phosphatase (EC_50_ 1.1 and 0.8 µLg/mL), respectively, compared to no or weak activity for the rest of the isolated furanocoumarins [142]. The effect of *A. sinensis* on many gynecological disorders is well-known; however, this effect is usually imparted through the hormonal-like activity of this herb, which raised many concerns for its use in breast cancer patients by enhancing tumor growth [143].

### 4.14. Skin Whitening

The root extract of *A. dahurica* was investigated on NK-1R and Wnt/*β*-catenin signaling, and evaluated against NK-1R on melanogenesis in B16F0 cells. The extract showed a potent reduction in Neurokinin-1 receptor and Wnt/*β*-catenin signaling activities *via* reducing the expression of *β*-catenin, MITF, LEF-1, TYR, TRP1, and TRP2, and enhancing the expression of GSK3*β* [144].

### 4.15. Immunomodulatory Activity

A polysaccharide isolated from *A. dahurica* exhibited an immunoregulatory activity in a Zebra fish model. It activated phagocytosis, enhanced the production of (NO), and promoted the secretion of (IL-6, IL-1*β*, and TNF-*α*) [90].

### 4.16. Effect on Gut Flora

The aqueous extract of *A. dahurica* containing novel polysaccharides was evaluated for its interaction with gut flora. The pure polysaccharide was administered at a dose of 200 mg/kg daily to mice for 21 days. The microbial composition was evaluated in fecal samples using high-throughput sequencing. The polysaccharide showed an effect on the composition and structure of gut microbiota, and can potentially regulate the intestinal flora as prebiotics [145].

### 4.17. Insecticidal Activity

Essential oil isolated from *A. dahurica* and *A. pubescentis* roots were studied as pest management prospectives. When compared with *A. pubescentis*, *A. dahurica* showed better biting inhibition and insecticidal effects against *Ae. egypti* and *Stephanitis pyrioides*. In mosquito bioassays, components of the *A. dahurica* essential oil, 1-dodecanol and 1-tridecanol, showed antibiting activity against *Ae. egypti* [42].

Chung et al., 2012, investigated the immune toxicity effect of the essential oil from the leaves of *A. anomala*, *A. cartilagino-marginata* var. distans, *A. czernevia*, *A. dahurica*, *A. decursiva*, *A. fallax*, *A. gigas,* and *A. japonica*. Among them, the essential oil of *A. dahurica* showed a significant toxic effect against early fourth-stage larvae of *Ae. aegypti* (LC_50_ 43.12 ppm) [146]. In another study, out of 33 plant species tested, Champakaew et al., 2015, found that *A.sinensis* essential oil showed the best repellent activity against *Ae. egypti* with a complete protection time of 7.0 h [53].

## 5. Discussion

In this review work, genus *Angelica*’s various phytochemical constituents, folk medicinal uses, adverse effects, potential toxicity, and reported biological activities have been summarized, with a special emphasis on *Angelica dahurica*. The *A. dahurica* species was selected for more detailed literature reviewing, as it is one of the most important and popular members of genus *Angelica,* being rich in coumarins and furanocoumarins, as one of the highly important class of compounds accounting for a wide range of biological activities *viz.* analgesic, anti-inflammatory, anti-coagulant, and cytotoxic activities. *Angelica dahurica* is commonly reported in folk medicinal practices, such as its use against skin diseases, pruritus, common cold, headache, and toothache; hepatoprotection; its antimicrobial effect; and its anti-inflammatory and cytotoxic activities. Different phytoconstituents were summarized covering different chemical classes, including coumarins, furanocoumarins, phtalides, polysaccharides, benzofurans, polyacetylenes, and essential oils. A total of 64 essential oil and other volatile components were reported from different *Angelica* species, together with 32 coumarins and furanocoumarins, six phthalides, six polyacetylenes, two benzofurans, and two polysaccharides. Essential oil components were traced from different *Angelica* species *viz. A. archangelica*, *A. archangelica* subsp. *Archangelica, A. dahurica, A. pubescentis, A. sinensis, A. glauca, A. gigas, A. acutiloba, A. major, A. urumiensis, A. pancicii, A. viridiflora*, *A. cincta,* and *A. pubescens,* while coumarins, furanocoumarins, benzofurans, and polyacetylenes accumulated mainly in *Angelica dahurica*. Moreover, phthalides were reported from *A. gigas* and *A. sinensis*. Different biological activities were summarized and directly linked to the reported phytoconstituents. The analgesic, anti-inflammatory, anti-histaminic, anti-oxidant, and cytotoxic activities were mainly related to the richness of genus *Angelica* with coumarins and furanocoumarins. Furanocoumarins, especially isoimperatorin, imperatorin, and oxypeucedanin, showed strong acetylcholine esterase inhibitory activity, which explains their activity in CNS-related disorders and, furthermore, Alzheimer’s and dementia. Many oxygenated terpenoids had been reported from the essential oils of different organs of genus *Angelica,* thus carrying strong anti-oxidant and anti-microbial activities against a wide range of bacteria, viruses, and fungi.

## 6. Conclusions

In conclusion, this review summarizes the reported literature on the phytochemical and biological activities of genus *Angelica,* focusing on *A. dahurica* as one of the most important plant genera, with many folk medicinal uses. Genus *Angelica* is rich mainly in coumarins and furanocoumarins, with about 32 of them reported only from *A. dahurica,* followed by phthalides and polysaccharides. Such phytoconstituents showed many biological activities, including, mainly: antimicrobial, antioxidant, anti-inflammatory, hepatoprotective, insecticidal, antidiabetic, *etc*. This updated review provides valuable references regarding genus *Angelica,* thus enriching the scientific development and future research on genus *Angelica.*

## Figures and Tables

**Table 1:** Botanical characteristics of genus *Angelica*.

Plant Organ	Botanical Characteristics
Stem	Caulescent or acaulescent
	Solid or hollow
Leaf	Alternate
	Compound
	Pinnate
	Lanceolate shape
	Petiolate
	Exstipulate
Flower	Epigynous
	Small
	Long pedicels
Fruit	Dry
	Cremocarp (two mericarps)
	Mericarp is one-seeded

**Table 4:** Phthalides isolated from genus *Angelica*.

No.	Compound Name	Source	Reference(s)
1	*Z*-Ligustilide	*A. sinensis*	[86]
2	Senkyunolide A	*A. sinensis*	[86]
3	Butylphthalide	*A. gigas*	[86]
4	*Z*-Butylenephthalide	*A. gigas*	[86]
5	Butylidenephthalide	*A. sinensis*	[87]
6	Oxaspiroangelioic acids A, B, and C	*A. sinensis*	[88]

## Data Availability

Data are available upon request from the authors.
